# Optical Sensors Based on II-VI Quantum Dots

**DOI:** 10.3390/nano9020192

**Published:** 2019-02-02

**Authors:** Anna Lesiak, Kamila Drzozga, Joanna Cabaj, Mateusz Bański, Karol Malecha, Artur Podhorodecki

**Affiliations:** 1 Faculty of Chemistry, Wrocław University of Science and Technology, Wybrzeże Wyspiańskiego 27, 50-370 Wrocław, Poland; anna.lesiak@pwr.edu.pl (A.L.); kamila.drzozga@pwr.edu.pl (K.D.); 2 Faculty of Experimental Physics, Wrocław University of Science and Technology, Wybrzeże Wyspiańskiego 27, 50-370 Wrocław, Poland; mateusz.banski@pwr.edu.pl; 3 Faculty of Microsystem Electronics and Photonics, Wrocław University of Science and Technology, Wybrzeże Wyspiańskiego 27, 50-370 Wrocław, Poland; karol.malecha@pwr.edu.pl

**Keywords:** nanomaterials, colloidal quantum dots, sensors, detection mechanisms

## Abstract

Fundamentals of quantum dots (QDs) sensing phenomena show the predominance of these fluorophores over standard organic dyes, mainly because of their unique optical properties such as sharp and tunable emission spectra, high emission quantum yield and broad absorption. Moreover, they also indicate no photo bleaching and can be also grown as no blinking emitters. Due to these properties, QDs may be used e.g., for multiplex testing of the analyte by simultaneously detecting multiple or very weak signals. Physico-chemical mechanisms used for analyte detection, like analyte stimulated QDs aggregation, nonradiative Förster resonance energy transfer (FRET) exhibit a number of QDs, which can be applied in sensors. Quantum dots-based sensors find use in the detection of ions, organic compounds (e.g., proteins, sugars, volatile substances) as well as bacteria and viruses.

## 1. Introduction

Among the most interesting and promising nanomaterials are colloidal semiconducting quantum dots (QDs). These nanostructures have found already several commercial applications in displays [1], light concentrators [2], photovoltaics [3] and as optical probes in various bio-applications [4,5]. The main reasons for their still growing success are: broad absorption band (several hundreds of nm), narrow emission band (below 40 nm), high quantum yield (up to 95%), possibility of emission band tuning over a wide range of wavelengths (350–2000 nm) and high resistivity of optical properties on external physico-chemical conditions, e.g., pH, temperature or power of the excitation beam [6]. QDs have a high surface to volume ratio, which can be controlled by QDs size but also by the nanostructures shape. This high surface area equips them with much more functional groups compared to organic compounds [7]. This makes QDs much more reactive and thus more effective in biological sensing [8].

**Figure 1:**
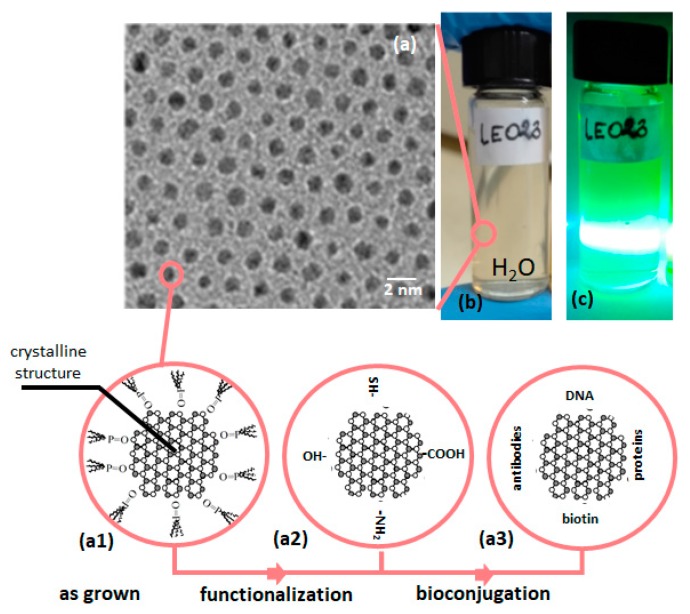
(**a**) TEM image of hydrophilic CdSe quantum dots. Schematic structure of selected quantum dot after synthesis (**a1**), after surface functionalization (with examples of most typical functional groups) (**a2**) and after bioconjugation (with examples of most common biomolecules used for detection/targeting) (**a3**). (**b**,**c**) Digital images of CdSe quantum dots dispersed in water with and without laser excitation.

The high quality QDs are typically grown as hydrophobic structures. In consequence, to make them useful for biological or medical application, additional post growth treatment is usually needed. This treatment includes QDs functionalization and in many cases bioconjugation (see Figure 1). One serious reason why QDs still do not dominate over organic markers (i.e., Green Fluorescent Protein, Rhodamine) lies in the absence of widely tested and already accepted protocols for QDs functionalization and bioconjugation [9]. There is also another reason to not use the QDs in biological sensing, especially in case of in vivo imaging, namely their toxicity. The QDs toxicity is a subject widely discussed recently in the literature [10]. The main reason affecting QDs toxicity is an aggregation of nanostructures in cells, organs, tissue etc. [11]. This is an even more serious problem than the chemical toxicity, due to dissociation of Cd atoms from CdSe/CdS QDs, and this is true for any types of nanostructures: semiconducting, dielectric or metallic nanostructures. Nevertheless, fortunately the QDs toxicity becomes a much less serious problem when the QDs are used for external sensing or for some in vitro applications when we can use their potential without barriers.

Concluding the above discussion, it can be seen that real benefits coming from extraordinary properties of QDs must be always compared to drawbacks of using inorganic probes in biological systems. In other words, for some specific applications QDs are an excellent choice, or very bad idea [12,13,14]. Among the applications where incontestably the advantages of QDs are utilized are sensing systems.

In this review, we present the main physical and chemical mechanisms used for detection of various species (bacteria, cells, nucleic acids, molecules, ions, etc.) with utilizing of QDs. We present the most successful examples of QDs applications in biology and medicine as optical and electrochemical sensors. Finally, we focus on the perspectives for further development in this field.

## 2. Comparison between Optical Properties of Organic Dyes and QDs

**Figure 2:**
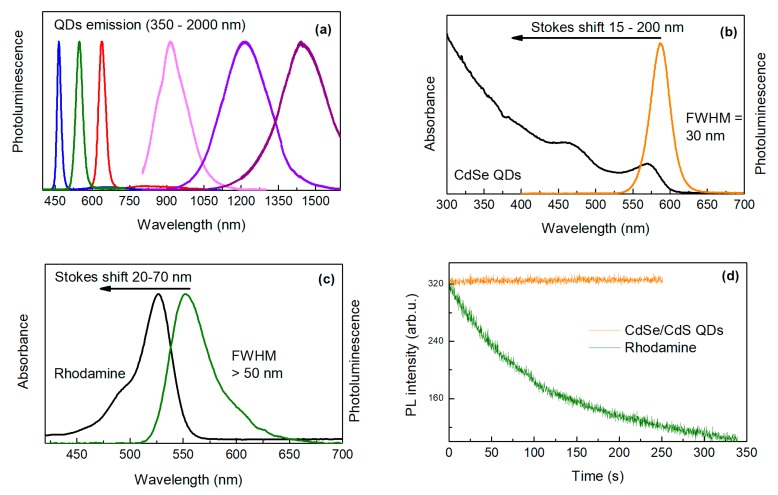
(**a**) Emission spectra from CdS QDs (left side) and PbS QDs (right side) with different size and chemical composition; (**b**) absorbance and emission spectra of CdSe/CdS quantum dots; (**c**) emission and absorption spectra of Rhodamine; (**d**) emission intensity vs illumination time for CdSe/CdS QDs and Rhodamine.

In comparison to organic dyes, QDs have the spectral position of absorption and emission dependent on their size (so-called Quantum Size Effect) [15]. During synthesis, this effect enables continuous tuning of the emission peak position in a wide range of wavelengths (Figure 2a). Moreover, the broad absorption of QDs allows free selection of the excitation wavelength and thus straightforward separation of the excitation and emission signal (Figure 2b,c) [16]. The fluorescence lifetimes of organic dyes are commonly too short for efficient temporal discrimination of short-lived autofluorescence of biological objects. In the case of QDs, the emission decay time can be tuned or selected with a proper choice of QDs composition (giving times up to several microseconds). This enables straightforward temporal discrimination of the signal from cellular autofluorescence and scattered excitation light by time-gated measurements, thereby enhancing detection sensitivity [17]. In contrast to conventional dyes, QDs emitting different colors (and functionalized with different groups) can be simultaneously excited by a single excitation wavelength. This makes QDs suitable for multiplex testing by simultaneously detecting multiple signals [18]. Moreover, QDs characterize with extremely high chemical stability and photostability (stability against chemical reactions induced by the incoming radiation). In addition, QDs are free from photobleaching [19] (Figure 2d) what is one of their most important advantages.

## 3. Fundamentals of QDs Sensing Phenomena

**Figure 3:**
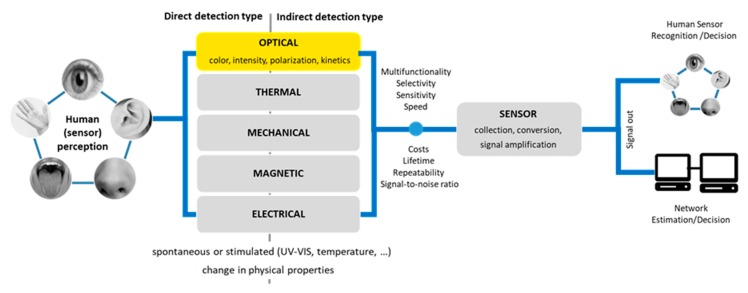
Signal processing characteristics for living organisms and sensor machines.

The unique optical properties of QDs make them attractive fluorophores that can be used both in vitro and in vivo in various biological studies, where traditional fluorescent labels based on organic molecules do not provide long-term stability, high enough intensity or where simultaneous detection of many signals is needed [7]. In sensors, the signal detection bases on a registration of the change in one of the physical properties (optical, thermal, mechanical, magnetic, electrical) of sensing material induced by the interaction with the analyte. Changing in optical properties of QDs like emission color, intensity, polarization or emission kinetics can be used as the principle in optical sensors system (Figure 3). In addition, obtained changes can be recorded directly by human senses or indirectly *via* the signal transformation, amplification, and visualization. All these factors determine sensors construction and their mechanism of action in the detection of various substances [20,21].

## 4. Basic Strategies for Analyte Detection

The use of QDs for sensors construction requires adjusting their optical properties adequately to the needs that arise their shape, size, the color of emission, position of the absorption band. Moreover, to get specificity of QDs in their sensing action, the surface modification—called functionalization—must be applied first [22,23]. Functionalization is the process of attaching, exchanging already attached chemical molecules present on the surface of quantum dots. Chemical and physical methods used for this purpose, include processes such as exchange of ligands, silanisation, the creation of additional coatings or dendrimeric structures [24]. The presence of ligands at the QDs surfaces affect their size, shape and physico-chemical properties, e.g., surface charge and chemical reactivity. Surface modifications allow the control of colloidal stability of QDs and their dispersion in non-polar environments (organic solvents in which they are most commonly synthesized) and polar (e.g., water, in which solubility is necessary for biological and medical applications). Moreover, the surface attached ligands determine the possibility of QDs conjugation to biological molecules (bioconjugation) or to determine their potential in applications where QDs must be embedded within the matrix [25,26].

In order to achieve high selectivity of QDs sensor, QDs are coupled to various vectors specific for an analyte. Wales et al. constructed a sensor for the selective detection of dicofol, a substance used to kill mites. For this purpose, they used CdS QDs with glutathione on their surface, whose both amino- and carboxyl- functional groups interact with chloride groups in the dicofol structure, thus leading to an increase in fluorescence intensity, which was directly proportional to the dicofol concentration in the studied sample [27,28].

**Figure 4:**
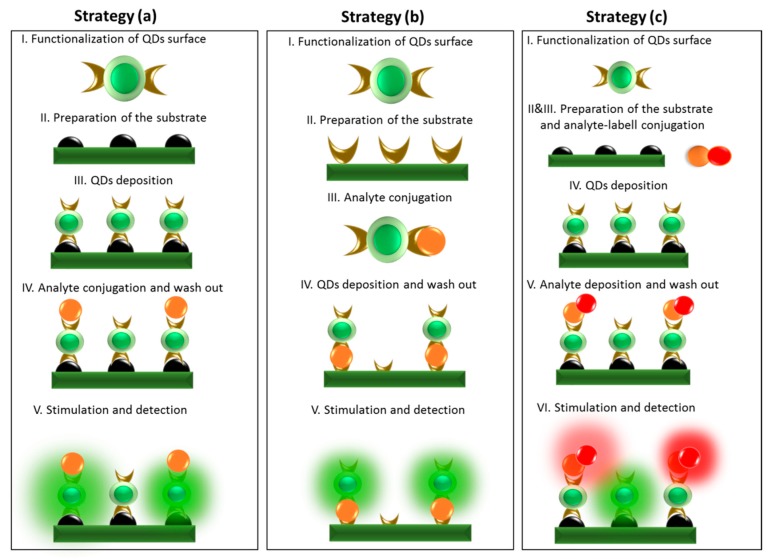
Three examples of the strategy of QDs-based optical sensors (strategy **a**—modification of substrate with QDs directed to detection of analyte, strategy **b**—modification of substrate for detection of analyte-QDs complex, strategy **c**—using the analyte labeled with appropriate fluorophore).

The QDs-based sensors can be designed in several ways, depending on demands regarding their sensitivity, types of detected analytes, costs or complexity of their preparation. Figure 4 shows the examples of preparation protocols used in QDs-based optical sensors.

In all cases, the protocol starts with the appropriate modification of QDs surface selectivity. As a result, QDs are targeted to determine a particular analyte. An important aspect is also the preparation of substrates which can take an active part in the detection protocol. Strategies (a) and (b) differ in Stages III and IV, which occur in reverse order. While in Strategy (a) Step III is the deposition of QDs, in Strategy (b) it is Step IV. This stage can be made using methods such as layer-by-layer [29], sol-gel [30] or electrochemical method [31]. Stage IV in Strategy (a) and III in Strategy (b) are a conjugation of the analyte, which may be possible thanks to the previously prepared and targeted substrate. Jie et al. proposed the coupling of the analyte with the previously prepared substrate, based on CdSe nanocomposites, using antibodies selective for an antigen called human IgG [32].

The final step in all strategies is QDs stimulation, which is used to detect the analyte. As a result, both the qualitative and quantitative assessment of the presence of the designated substance is possible. It is also possible to combine the first two strategies, resulting in Strategy (c), which uses the Förster resonance energy transfer between optical centers (QDs + QDs or dye).

The presented strategies differ in the number of steps that complicate detection and require a lot of user experience.

## 5. Physico-Chemical Mechanisms Used for Analyte Detection

**Figure 5:**
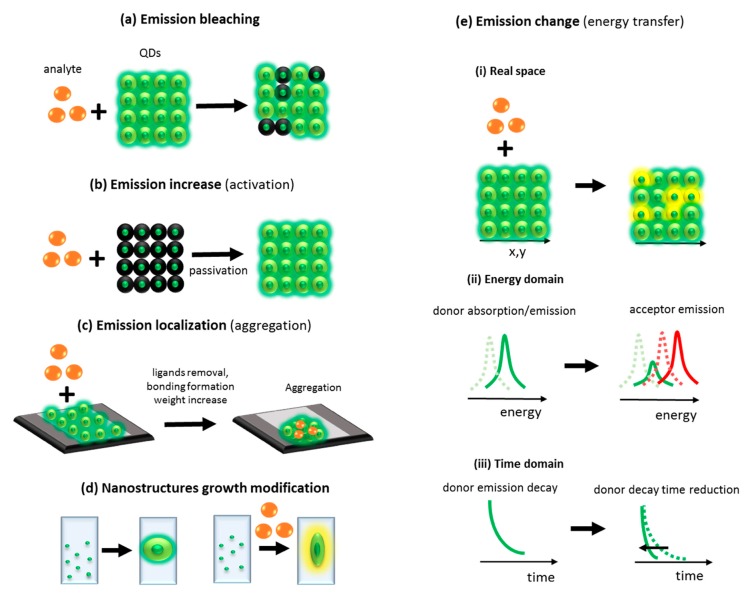
Examples of physico-chemical mechanisms used for analyte optical detection—emission bleaching (**a**), increase of emission (**b**), emission localization (**c**), nanostructures growth’s modification (**d**), emission change (**e**).

One of the most popular mechanisms used for detection of analyte relies on emission quenching from QDs. In this mechanism, due to the interaction of the QDs surface with the analyte, the QDs emission intensity decreases (Figure 5a) [33]. Another mechanism relies on an increase of QDs emission due to passivation of QDs surface by analyte (Figure 5b), e.g., addition of bovine serum albumin or nucleic acids resulted in increasing emission from CdS dots coated with mercaptoacetic acid [34].

The third mechanism, which can be used for analyte detection, is stimulated aggregation (Figure 5c). In this mechanism, due to an interaction of the analyte with the QDs surface, the surface ligands are detached and QDs aggregate. The aggregation can be also induced by analyte stimulated bonds formation between functionalized QDs [35].

There is also a very rarely used mechanism of analyte detection based on modification of the nanostructures’ growth process by introduction of the analyte during the nanostructures’ growth. Due to this perturbation, the nanostructures can have different emission or other properties which can be detected (Figure 5d). There is also the fifth mechanism commonly used for analyte detection based on changes in QDs optical properties. The changes come from excitation energy transfer from QDs to other optical center (QDs or dye). In consequences, the color of emission changes or emission decay time of donor is reduced (Figure 5e) [36,37].

### 5.1. Emission Bleaching

**Figure 6:**
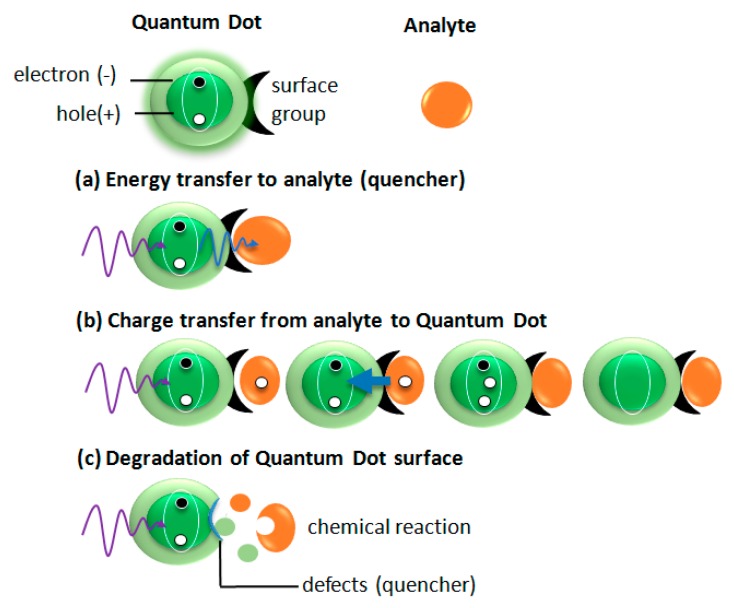
Examples of physico-chemical mechanisms responsible for quantum dots emission quenching—energy transfer from QD to analyte (**a**), charge transfer from analyte to QD (**b**), degradation of QD surface (**c**).

One of the basic mechanisms of an analyte detection is emission bleaching (Figure 5a). This mechanism can be induced by different physical phenomena schematically shown in Figure 6. In the first example, the absorption band of the analyte overlaps with the emission band of QDs. In such a case, the emission from QDs is absorbed by an analyte, which results in bleaching QDs emission. (Figure 6a). Another mechanism (Figure 6b) bases on a charge transfer from an analyte to QDs. When the additional electron or hole appears in an excited QDs the Auger processes lead to QDs ionization or charging. In both cases, the dot became non-emissive (dark). The third mechanism, which is responsible for QDs emission quenching under the interaction with the analyte is analyte-induced degradation of the QDs surface providing to emission decrease. As an example, thiol coated ZnS QDs in the presence of peptides showed a significant emission quenching [38].

### 5.2. Emission Change—Nonradiative Förster Resonance Energy Transfer (FRET)

When two optically active centers (donor and acceptor) are in close proximity to each other, (typically 1–10 nm) and an absorption spectrum of the acceptor overlaps an emission spectrum of the donor, the non-radiative transfer of the excitation energy from the donor to acceptor appears. This phenomenon is called FRET (Förster Resonance Energy Transfer). For the experiments using the FRET approach, photostable emitters must be used, characterized by a quantum efficiency greater than 0.1 and a high brightness (one in which the absorbance coefficient Ɛ is greater than 50.000 M^−1^·cm^−1^) [39]. All these conditions are perfectly fulfilled by the quantum dots. The effectiveness of FRET is inversely proportional to the distance between the donor and acceptor and defined as:

(1) $$K_{FRET}=\frac{1}{1+{\left(\frac{R}{R_{0}}\right)}^{6}},$$

where: *K_FRET_*—FRET efficiency; *R*—distance between donor and acceptor; *R_0_*—distance at which the FRET efficiency is equal to 0.5 [40].

The highest sensitivity of FRET signal is for a distance between the donor and acceptor in the range from 0.5R_0_ to 1.5R_0_ [40]. For years researchers have been using the FRET mechanism to monitor intracellular interactions, due to its sensitivity to molecular rearrangements in the 1–10 nm range (this is the scale correlating with the size of biological macromolecules and the possibility of creating bonds between them) [41].

**Figure 7:**
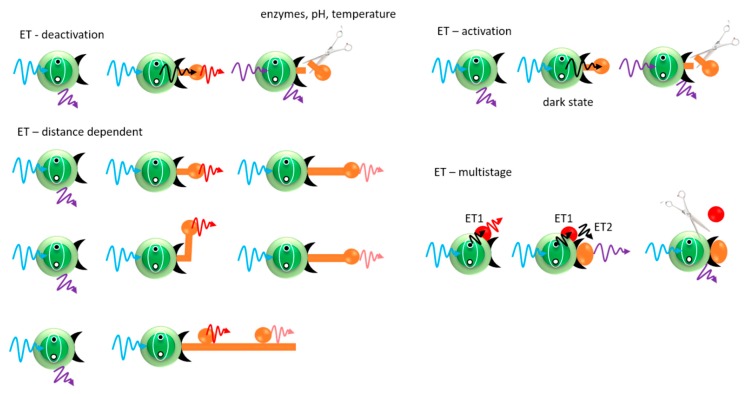
Different detection possibilities with use of nonradiative energy-transfer phenomena.

The universality of FRET method allows its use in nanosystems as well [42]. FRET yield is typically measured by observing one of the three parameters of the fluorescent donor: fluorescence intensity, spectral response or average fluorescence lifetime. Moreover, FRET has found application in many sensing systems giving the possibility of applying it to three analyte detection strategies. Figure 7 shows different processes which can be detected with use of FRET. The first mechanism uses analyte as optically active acceptor. In this case, the analyte attachment as well analyte removal can be observed as a change in the optical signal. The other strategy uses the analyte as the emission quencher and was also discussed in the previous paragraph [43]. The third strategy is more complex and uses a multistep energy-transfer phenomenon. Detection using FRET between the QDs, as donors, directed to a linker with an acceptor, associated, e.g., with a receptor protein, is widely used to study the receptor-ligand interactions and changes in protein conformation after binding to the target analyte [44]. Thanks to this, in analytics consisting of several acceptors, QDs can interact with only one of them, which significantly improves the efficiency and sensitivity of the FRET method [45].

### 5.3. Analyte Stimulated QDs Aggregation

The colloidal QDs solution is sensitive to the presence of additional charges either on QDs surface or in the solvent, which may result in QDs aggregation. The charges may be introduced or induced by an analyte, which ultimately is manifested by QDs aggregation [46,47,48]. In the absence of analyte, a fluorescence comes from the whole volume of the QDs solution, while after aggregation caused by the analyte, the emission is localized [19]. This type of stimulation belongs to qualitative tests.

## 6. Photoelectrochemical and Electrochemical Methods of Analyte Detection

The chemical detection method is usually signaled by the following ways: competition binding assay of labelled and unlabeled analytes, using labelled molecules specific for immobilized analytes, sandwich formation or enzyme immunoassay, where enzymatically active substrate is added that changes color or fluorescence after interaction with enzyme-related analyzes [49].

Mo et al. [50] used a redox mechanism in the detection of hydroquinone in water samples. They have observed that ZnS QDs cannot react with hydroquinone. When hydroquinone and K_2_S_2_O_8_ were added into ZnSe QDs solution, no new photoluminescence (PL) peak was observed. Comparing with the pure ZnSe QDs solution, the PL intensity of the mixture decreased. This result reveals that hydroquinone oxidation product can efficiently quench the fluorescence emission of ZnSe QDs by energy transfer in electrochemiluminescence mechanism.

The development of new, reliable, fast and efficient methods for detecting anthropogenic and natural substances, both organic and inorganic, is a huge challenge for modern analytical chemistry and diagnostics. An alternative to such methods are electrochemical strategies using semiconducting quantum dots. The growing interest in the construction of electrochemical devices using quantum dots results from their aforementioned properties. Due to these features, small changes in the external environment lead to great changes in particle properties and electron transfer. Based on these significant changes, quantum dots are prone to engaging in heterogeneous redox chemistry with the surrounding environment. QDs are also used as carriers of biomacromolecules in bioanalytics. For this purpose, the chemical functionalization of QDs is carried out by means of a functional cap layer that allows the molecules to be trapped. The immobilization of biomolecules (e.g., the enzyme catalyzing the redox reaction) on the surface of semiconductor QDs causes QDs to promote direct electron transfer between biomolecules and the surface of the electrode, which significantly affects the operation of the system by enhancing the sensitivity due to signal amplification. A tremendous increase of development of electrochemical sensors based on QDs has been observed over the past decades due to the simplicity of implementation, high selectivity, and specificity of the system, low cost and the possibility of miniaturization [51]. Moreover, research carried out by Bard et al. revealed that CdS QDs could also act as multi-electron donors or acceptors at a given potential due to trapping of holes and electrons within the particle [52]. On the other hand, the surface structures of QDs also play a key role in determining the properties of the particles [53].

Liu et al. [54] described an electrochemical assay strategy for specific recognition of tumor cells. For this purpose, gold nanoparticles (AuNPs) have been assembled onto the indium tin oxide (ITO) substrate to create a specific, biocompatible interface to effective capture of tumor cells. CdSe/ZnS QDs labelled on the cell surface have been used as an amplified signal during the square wave stripping voltammetry (SWSV). The developed biosensing platform shown good analytical performance with a broad linear range, good selectivity and low limit of detection (LOD).

Electrochemiluminescence (ECL) is a method which aims to convert electric energy into radiation energy, in which electrochemically generated intermediate products undergo a high energy electron transfer reaction to generate excited states, resulting in the emission of a measurable luminescence signal [55]. As a form of luminescence (light emission without heat), ECL is characterized by the fact that light emission occurs when an appropriate potential is applied to the electrode, as a result of which the oxidation or reduction reaction takes place. There are several features that distinguish ECL from other techniques, e.g., chemiluminescence (CL). It is clear that the electrochemical reaction that takes place allows for precise time control. This means that the emission of light can be delayed to the desired moment, e.g., an immune reaction or an enzymatic reaction. Another advantage of ECL is the ability to control the location of the reaction, which means that there is the possibility of limiting the emission of light to a specific area relative to the detector. Electrochemiluminescence may occur as a result of two independent processes: annihilation of ions and co-reactant ECL. The annihilation of ions consists in creating states of excited molecules due to the transfer of electrons between radical ions on the surface of the electrode. The ECL co-reactant is due to the use of anode or cathodic potential in a solution containing phosphor and co-agent molecules. Depending on the potential application, the phosphor or co-reactant molecules can be reduced or oxidized to form radical ions and medium compounds, followed by decomposition and formation of excited states that cause light emission [56].

## 7. Applications of QDs-Based Sensors

### 7.1. Detection of Ions

Fast and reliable detection and recognition of ions in the environment is extremely important in modern medicine and environmental protection. Among the ions, heavy metal ions such as mercury, cadmium and lead due to their high toxicity and negative health effects (cardiovascular diseases, cancer, liver, kidney and central nervous system disorders, reproductive and neurological disorders) require constant control concentration and rapid response in view of its possible reduction [57,58,59,60,61].

Hydrophilic QDs have been demonstrated to be a promising sensor probe for fluorescence-based sensing of heavy metal ions [57] such as Pb^2+^ [62], Cd^2+^ [63], Cu^2+^ [64], Hg^2+^ [65], Fe^3+^ [62], etc. Table 1 shows an exemplary strategies for heavy metal ions determination with using of QDs.

Detection of ions with the use of photoluminescent-induced changes in the QDs involves the use of a number of ligands—derivatives of thioalkyl, mercaptoacetic or dihydrolipoic acids. The affinity of the thiol group to QDs results in self-assembly of the ligands on the surface of the dots, as a result of which the hydrophilic carboxylic groups are exposed on the surface towards the surrounding aqueous solution [6]. Chen and Rosenzweig proposed a method for detection of Zn^2+^ and Cu^2+^ ions. They exploit the fact that surface-modified QDs with mercaptoacetic acid show high sensitivity and selectivity to Cu^2+^ copper ions present in the mixture. The result of adding Cu^2+^ to the ligand-QDs complex is a reduction of the intensity of PL QDs. Such constructed sensor exhibited high LOD 0.8 μM [13]. Selective quenching PL was also used by Li et al. [58] They constructed sensor sensitive to the presence of mercury ions Hg^2+^ In this measuring system, CdSe/ZnS QDs have been modified with sulfur calixarene (S-Calix). The linear range of this system was found as 0–3 × 10^−5^ M with a LOD 15 nM.

Zhou et al. [57] presented a ratiometric fluorescence sensor for real-time and on-site detection of Fe^3+^ ions based on CdTe QDs-doped hydrogel optical fiber with a broad linear range from 0 to 3.5 μM and high LOD 14 nM. The ratiometric configuration of the proposed sensor provides a built-in calibration to eliminate the analyte-independent interferences. Two types of CdTe QDs, which possessed different emission bands, have been synthesized for ratiometric measurements. One of the QDs, coated with thioglycolic acid, exhibits green emission and is insensitive to metal ions, thus serving as a reference. The other QDs as the specific recognition element, coated with N-acetyl-l-cysteine, are red emissive and show high selectivity of fluorescence quenching towards Fe^3+^ ions. To avoid mutual interference, the green emissive QDs and red emissive QDs are doped in discrete sections of the hydrogel optical fiber. As a result, it has been observed a decrease in PL intensity.

### 7.2. pH Detection

Among the group of chemical sensors, the pH sensor is the object of greatest interest of scientists, because pH is one of the most important parameters in biochemical industrial processes [66,67,68,69,70]. Properly modified QDs, using organic ligands, may gain sensitivity to changes in pH. This property has a promising application in the design of a variety of luminescence sensors, examples of which are shown in Table 2.

In the work of Tomasulo et al. [66], the adsorption of pH-sensitive 1,3-oxazine on the surface of CdSe/ZnS QDs gives the possibility of changing the luminescence of inorganic nanoparticles by means of chemical stimulation: 1,3-oxazine rings open in an acidic or basic environment to form nitrobenzyl phenolate chromophores. This transformation activates the energy-transfer path from excited quantum dots to ligands and facilitates energy transfer in the opposite direction. As a result, the intensity of PL QDs decline. Such a system can be used in aqueous solutions for pH changes in the range of 3–11.

The Snee group proposed a sensor based on signal transduction by FRET between the QDs and a fluorescent pH-sensitive squaraine dye attached to the surface of the QDs. The detection system process consisted in modulating the FRET efficiency resulting from the overlap of the absorption spectrum of squaraine with the emission of QDs. The emission of QDs (donor) was inhibited by the presence of squaraine, acting as an acceptor. Lowering the pH value caused a rise in the photoluminescence intensity of QDs [67].

Many works present pH-sensitive sensors that use semiconductor QDs combined with thiol compounds [6]. The emission of QDs fluorescence with mercaptoacetic acid on the surface allowed in vitro detection (but only in an acidic environment) and in live cells. The increase in intracellular pH has given an increased intensity signal of PL QDs [12]. In contrast, QDs combined with mercaptosuccinic acid (MSA) proved to be a simple system for the detection of urea. Hydrolysis of urea generates hydroxide anions, gradually raising the pH of the solution. With increasing urea concentration, the intensity of PL QDs increased [70].

### 7.3. Detection of Organic Compounds

#### 7.3.1. Proteins

Proteins are among the most important biomolecules found in the body. In addition to the basic building, transporting and regulating functions, proteins also act as biological catalysts–enzymes. The function of proteins is also invaluable in the immune system-acting as immunoglobulins. Due to the extremely important functions of proteins, it is necessary to monitor their concentration and the processes in which they take part [19,71,72,73,74,75]. QDs-based FRET nanosensors have been developed to monitor a variety of enzymes including alkaline phosphatase, ATPase renin, protein kinase, DNA methyltransferase, DNA glycosylase, and telomerase [76]. A different strategies for determination of proteins are presented in Table 3.

Xu et al. have presented a novel label-free fluorescent assay for monitoring the activity and inhibition of protein kinases based on the aggregation behavior of unmodified CdTe QDs with very high LOD 5.0 fM. In this assay, cationic substrate peptides induce the selective aggregation of unmodified QDs with an anionic surface charge, whereas phosphorylated peptides do not. Phosphorylation by kinase alters the net charge of peptides and subsequently inhibits the aggregation of unmodified QDs, causing an enhanced QDs fluorescence [19].

Lv et al. [71] have proposed detection of C-reactive protein (CRP) based on fluorescence changes by CdSe/ZnS QDs, where QDs surfaces were modified with monoclonal antibodies. The fluorescence intensity has increased with the increasing of antigens concentration. The assay for the detection of CRP can provide a wide analytical range of 1.56–400 ng/mL with the LOD 0.46 ng/mL and the limit of quantification = 1.53 ng/mL.

Another example of a protein biosensor was developed by Zhang group. Using DNA-ZnS:Mn^2+^ QDs as the energy donor and WS_2_ as the energy acceptor. DNA-ZnS:Mn^2+^ QDs were hybridized with biotin-DNA to obtain dsDNA. When Exonuclease III was added into the system, the biotin-DNA was hydrolyzed for the stepwise removal of mononucleotides from the 30-hydroxyl termini of dsDNA, releasing DNA-ZnS:Mn^2+^ QDs into solution. After incubation with WS_2_ nanosheets, DNA-ZnS:Mn^2+^ QDs were absorbed on the surface of WS_2_ due to their stronger affinity towards ssDNA than that of dsDNA. As the results, the fluorescence intensity was reduced with the increasing concentration of Exonuclease III. There is a good linear relationship between the fluorescence intensities and the concentration of SA (biotin-streptavidin) in the range of 5–150 ng/mL. The LOD was calculated as 2.8 ng/mL [72].

#### 7.3.2. Sugars

Among the most popular groups of biomolecules that are the object of scientists’ interest are sugars. Particularly noteworthy is glucose, the determination of which is important both in the pharmaceutical and food industries. Importantly, glucose monitoring is essential in the treatment of diabetes that is characterized by long-lasting hyperglycemia, making strict blood glucose control so important [77,78,79,80,81]. Table 4 shows an exemplary QDs-based sensors for sugars determination.

Monitoring of glucose in human blood and urine is essential for the diagnosis and treatment of diabetes. The Sarana group has developed a biosensor for the detection of glucose based on cadmium quantum dots with a thiol ligand on the surface. This system has been coupled with glucose oxidase-a catalyst for glucose oxidation reaction, which releases hydrogen peroxide [77]. In constructing this biosensor, a capture mechanism was used, involving the charge transfer. The electron released in the process of reducing H_2_O_2_ to O_2_ has been moved towards the exciton of QDs, acting as an acceptor. As a result of this process, a QDs-ion was formed and decreases of fluorescence intensity were observed [82].

Another sensing method of glucose approach is based on FRET between CdTe QDs as an energy donor and gold nanoparticles (AuNPs) as an energy acceptor. The specific combination of concanavalin A(ConA)-conjugated QDs and thiolated-cyclodextrins (b-SH-CDs)-modified AuNPs assembles a hyperefficient FRET nanobiosensor. In the presence of glucose, the AuNPs-b-CDs segment of the nanobiosensor is displaced by glucose which competes with b-CDs on the binding sites of ConA, resulting in the fluorescence recovery of the quenched QDs. Experimental results show that the increase in fluorescence intensity is proportional to the concentration of glucose in a linear range of 0.10–50 µM under the optimized experimental conditions. In addition, the sensor has high sensitivity with a LOD as low as 50 nM, and has excellent selectivity for glucose over other sugars and most biological species present in serum [78].

Riedel et al. [79] investigated the light-triggered reaction of the redox molecules, hexacyanoferrate, and ferrocenecarboxylic acid, at CdSe/ZnS quantum dot modified gold electrodes for light-driven applications. Here, electron transfer between QDs and redox mediators has been found to be feasible. Additionally, photoluminescence measurements in solution demonstrate the strong interaction between the QDs and the redox species by quenching of QD fluorescence. Subsequently, the established QD–mediator systems have been combined with the enzymes, pyrroloquinoline quinone-dependent glucose dehydrogenase and fructose dehydrogenase, to the feasibility of electrically contacted enzyme/QD biohybrids. This demonstrates the photoelectrochemical principle displays applicability for sensing and for driving QD electrodes by biocatalytic sugar consumption.

#### 7.3.3. Nucleic Acids

One of the most important biomolecules is deoxyribonucleic acid (DNA), which is responsible for determining inherited traits and storing genetic information necessary for the replication of living organisms. The sensors that use DNA molecules are a great tool not only to detect individual DNA or RNA molecules but also molecules belonging to other classes of biomolecules [83,84,85,86]. Nucleic acids do not have properties that would be useful for their direct detection, so their detection requires the use of, e.g., fluorescent markers [14,87]. Table 5 shows an exemplary QDs-based sensors for nucleic acids determination.

Nejdl et al. reported a systematic study of the self-assembly of CdTe QDs stabilized by mercaptosuccinic acid (MSA) These QDs were used for the preparation of a fluorescent (off–on) probe based on methylene blue as a quencher for the specific determination of nucleic acid from urine. Using this technique, it was possible to determine the DNA isolated from the urine and decide whether the amount of DNA was in an acceptable range. The LOD was calculated as 0.003 μg·mL^−1^ DNA. Such constructed sensing systems can be used for very sensitive detection of DNA [83].

Mohammadinejad group has prepared mercaptosuccinic acid-capped CdTe quantum dots, which were successfully fabricated as a simple synthesized and sensitive fluorescence sensor for tandem determination of mitoxantrone and ribonucleic acid and also monitoring their interaction. Due to the adsorption of positively-charged mitoxantrone on the surface of negatively-charged quantum dots through electrostatic interactions, the fluorescence intensity of mercaptosuccinic acid-capped CdTe QDs can be effectively quenched by mitoxantrone. After addition of ribonucleic acid to mitoxantrone–QDs solution, mitoxantrone mainly bound to the uracil (C=O) and adenine (C=N) sites of ribonucleic acid. A complex which was formed between mitoxantrone and ribonucleic acid, prevented more interactions between quantum dots and anticancer drug resulted in enhancing of fluorescence intensity. Quantitative results were obtained for all combinations with a linear range of 20–10,000 pM and a LOD of 3–52 pM [87].

#### 7.3.4. Neurotransmitters

Neurotransmitters play a key role in acting as mediators of the autonomic system in the human body. Detection of biological abnormalities related to neurotransmitters (their concentration or metabolites) in the biological fluid is of fundamental importance in medical diagnostics. Differences in the level of neurotransmitters may be related to the occurrence of various diseases substrates such as schizophrenia, Parkinson’s disease, Alzheimer’s disease, Huntington’s chorea, adrenocortical cancer and other cancers and depression. Monitoring the concentration and products of the neurotransmitters synthesis pathway is a promising strategy for early detection and thus preventing the development of these diseases. Table 6 shows an exemplary strategies based on QDs for neurotransmitters determination [88,89,90,91,92].

Wang et al. [89] described a fluorescence assay for the fluorometric determination of dopamine (DA). It is based on the use of silica-coated CdTe quantum dots (QD@SiO_2_). When dopamine is added to a solution of the QD@SiO_2_ and then oxidized by oxygen under the catalytic action of tyrosinase to form dopamine quinone, the fluorescence of QD@SiO_2_ decreases, due to an electron transfer quenching process. Linear relationship over the range from 0.05 to 30 μM DA and high LOD of 12.5 nM. This suggested that the novel assay provided a promising possibility for further utilizing as an efficient platform for measuring DA in biological and environmental applications.

Another example of dopamine detection prepared by Hun et al. described photoelectrochemical sensor for dopamine which yields a signal upon irradiation with visible light. The electrons of SnSe QDs were excited under irradiation with visible light and transformed from valence band to conduction band. Dopamine, as an electron donor, provided the electrons to SnSe QDs. As a result, the enhanced photocurrent was obtained. This sensing system responds linearly to DA in the 0.01 μM to 10 μM concentration range and with a 3 nM LOD [90].

A novel molecular imprinted sensor based on CdTe@SiO2 QDs has been developed by Wei et al., for norepinephrine (NE) recognition. The synthesized nanosensor had a distinguished selectivity and high binding affinity to NE. Under optimal conditions, the relative fluorescence intensity of CdTe@SiO_2_@Molecular-imprinted-polymer linearly decreased with an increase in the concentration of NE in the range of 0.04–10 μM. The LOD was calculated as 8 nM [91].

A very promising alternative for construction of new micro-devices is LTCC (Low Temperature Co-fired Ceramic [93]) technology, consisting in the creation of three-dimensional structures of electronic systems based on pressed and co-poured ceramic foils with printed functional layers [94,95]. This method was used by Baluta et al. They proposed a convenient fluorescence dopamine-sensing strategy based on polydopamine formed on the surface of graphene quantum dots (GQDs). This sensing system utilized the catalytic oxidation of DA to dopamine-o-quinone (DOQ), and then to poly(DA), which can selectively quench the strong luminescence of GQDs due to FRET. Such constructed biosensor exhibited a broad linear range from 1 μM up to 200 μM with LOD 80 nM [96].

#### 7.3.5. Pesticides

Pesticides are chemical compounds increasingly used in agricultural production, which play an important role in ensuring optimal efficiency and maximizing income. Despite the positive impact on economic aspects, excessive use of pesticides can lead to the production of harmful chemical intermediates in vegetables, fruits and other agricultural products, which is a serious threat to food safety and human health. In order to ensure constant control of the concentration of these substances in agricultural products, it is necessary to find quick methods to confirm the presence and define their concentration [97,98,99,100,101,102]. Table 7 presents an exemplary fluorescent strategies for pesticides determination with using of QDs.

Kanagasubbulakshmi et al. [99] show the thioglycolic acid (TGA) capped CdTe QDs were highly dispersed and uniform in nature. The TGA surface modification of CdTe did not lead to the agglomeration of QDs. But when an interaction occurred with malathion, the aggregation was formed due to the functional group detachment. The linearity was obtained in the range of 3–21 nM with the LOD 0.68 nM. While Jiménez-López et al. [97] has proposed a multi-commutated flow analysis method for the determination of glyphosate, based on the quenching effect produced by this herbicide on the fluorescence of CdTe quantum dots with the LOD 0.52 μg·mL^−1^.

Another example of a sensitive direct competitive biomimetic immunosorbent assay method for pesticide detection was proposed by Liu et al. [98], using the hydrophilic imprinted film as artificial antibody and CdSe/ZnS QD label as a marker. A decrease in the fluorescence of the CdSe/ZnS QD conjugate was observed when QDs has attached to the Trichlorfon. Under optimal conditions, the LOD and sensitivity of the biomimetic immunosorbent assay method were found as 9.0 μg·L^−1^ and 5.0 mg·L^−1^.

#### 7.3.6. Toxins

Among the biggest threats to our community are toxins, whose quick and sensitive detection in aqueous solutions, body fluids, food, and drinking water enables the immediate application of appropriate remedies. Among the most common sources of toxins are bacteria whose toxins can be detected in trace amounts in urine or blood after poisoning. However, saliva and nasal swabs can be tested to confirm exposure to toxins even before the onset of symptoms. Another source of toxins in the environment is industrial, military or agricultural activity. Pollutants, such as pesticides and residues from explosives, pollute soil and groundwater and can easily enter the human body [103,104,105,106,107]. Table 8 shows an exemplary QDs-based sensors for toxins determination.

A new type of molecularly imprinted silica layers appended to CdS/CdSe/ZnS QDs (MIP-QDs) for saxitoxin were fabricated through the surface grafting technique. Sun et al. demonstrated that the synthesized MIP-QDs exhibited excellent selective fluorescence quenching to saxitoxin because of the complementary imprinted cavities on the surface of MIP-QDs. Such constructed MIP-QDs sensor exhibited excellent linearity in the range of 20.0–100.0 μg/L with LOD 0.3 μg/kg [103].

Wang et al. [104] reported a novel FRET-based nanobiosensor that uses luminescent QDs and dark quencher-labelled peptide probes to rapidly (on the order of hours) detect and quantify biologically active Botulinum neurotoxin (BoNT) and differentiate serotypes A and B, which is based on quantifiable differences in the photoluminescence (PL) intensity of QD reporters. The biorecognition elements for these probes are peptides that contain an amino acid sequence specific for BoNT/A or /B cleavage, a poly(histidine) sequence at the C-terminal for assembly on the QDs, and a dark quencher label (a dye with no native fluorescence) that quenches the QD PL only when the peptide chain is uncleaved (i.e., in the absence of the target BoNT). The sensor signal scaled linearly with the analyte concentration over a range of 8–200 nM, with 4 pM as the LOD.

#### 7.3.7. Volatile Substances

Volatile organic compounds (VOCs) are organic chemical compounds with an evaporation temperature close to room temperature. These substances are commonly used in industry and as household products. Too high exposure to volatile organic compounds can have both short- and long-term adverse effects on health, such as respiratory failure [108,109,110,111]. An exemplary QDs-based sensors for volatile substances determination are presented in Table 9.

Liu et al. [109] has presented work in which PbS-QDs/TiO_2_-nanotubes arrays (PbS QDs/TiO_2_ NTARs) are prepared by successive ionic layer adsorption and reaction, which are used to fabricate the gas sensor. The gas sensing performance shows that PbS QDs/TiO_2_ NTARs possess a good response towards ammonia gas at room temperature. The enhanced sensing mechanism lies in the fact that PbS QDs in PbS QDs/TiO_2_ NTARs may provide more sites to absorb the ammonia molecules and increase the depletion layer. The well-combined interface may provide effective transportation of the electrons as well as the direct transportation of the electrons along the TiO_2_ NTARs axis. This sensing strategy exhibited linearity in the range from 2 to 100 ppm at room temperature, with a LOD 2 ppm.

Barroso et al. [110] presented a new strategy for the detection of methanol using fluorescence spectroscopy and photoelectrochemical (PEC) analysis. The analytical system is based on the oxidation of cysteine (CSH) with hydrogen peroxide (H_2_O_2_) enzymatically generated by alcohol oxidase (AOx). H_2_O_2_ oxidizes capping agent CSH, modulating the growth of CSH-stabilized CdS QDs. Disposable screen-printed carbon electrodes (SPCEs) modified with a conductive osmium polymer (Os-PVP) complex were employed to quantify resulting CdS QDs. This polymer facilitates the “wiring” of in situ enzymatically generated CdS QDs, which photocatalyzed oxidation of 1-thioglycerol (TG), generating photocurrent as the readout signal. As a result, an increase of the fluorescence intensity was observed.

Sotelo-Gonzalez et al. [111] has prepared colloidal Mn^2+^-doped ZnS nanoparticles exhibiting room temperature phosphorescence (RTP) emission and water solubilized by capping the QDs surface with l-cysteine. Such coating of the nanoparticle with cysteine groups allows their analytical application for acetone determination (selected as model ketone species) in aqueous media (by measuring the quenching on the RTP emission of such QDs after direct interaction with the analyte). It was observed that the rise of acetone concentration efficiently quenches of the phosphorescence emission. The linear range of the developed methodology turned out to be at least up to 600 mg·L^−1^ with the LOD for acetone dissolved in an aqueous medium of 0.2 mg·L^−1^.

#### 7.3.8. Vitamins

Vitamins, which are found in many animal and plant tissues, play an essential role in proper metabolism and maintenance of body cells. Disorders in their synthesis or metabolism may be the cause of many serious diseases, hence the invention of sensitive and fast sensor devices is significant [112,113,114,115,116]. An exemplary QDs-based sensors for determination of vitamins are presented in Table 10.

Liu et al. [112] prepared a novel optosensing material based on quantum dots and graphene oxide for specific determination of Vitamin E. Ultra-high specific surface was obtained by synthesis of molecular imprinted polymer (MIP), which was stocked for specific Vitamin E reaction area. Under optimal condition, the fluorescence intensity of MIP was decreased linearly with the increasing concentration of Vitamin E. Such constructed sensor exhibited good linear range from 2.30 × 10^−2^–9.20 × 10^2^ μM with a LOD 3.5 nM.

Another strategy was used by Geszke–Moritz et al. [113] They used high fluorescence sensitivity to folic acid due to the high affinity of nitrogen atoms and carboxyl groups to doped QDs. Due to the quenching of fluorescence intensity QDs, it is possible to detect folic acid concentrations from LOD 11 μM.

Ganiga and others presented an optical sensor that uses FRET for fast and sensitive detection of ascorbic acid (AA). For this purpose, CdS QDs and diphenylcarbadiazone (DPCD) were used. In the presence of AA, the DPCD was transformed into diphenylcarbazide (DPC), which resulted in the recovery of fluorescence. Changes in fluorescence intensity enabled the detection and determination of AA concentration in the linear range of 60–300 nM with LOD 2 nM [116].

### 7.4. Detection of Bacteria and Viruses

The identification of pathogenic bacteria and viruses in food, water, air, and body fluids is extremely important because of their drastic impact on our society. The result of the human body’s contact with pathogenic bacteria or viruses is serious gastrointestinal infections that can lead to patient death without a doctor’s control. Importantly, pathogenic bacteria also produce toxins that are responsible for the occurrence of serious diseases, such as hemorrhagic colitis, characterized by painful abdominal cramps and bloody diarrhea or hemolytic-uremic syndrome, the most severe effect of which is an acute renal failure. Fast and sensitive detection of pathogenic bacteria and viruses is necessary to prevent the occurrence of epidemics or severe forms of the disease [117,118,119,120,121]. Table 11 shows an exemplary strategies for bacteria and viruses determination.

Xue et al. [120] presented a novel fluorescent biosensor for ultra-sensitive and rapid detection of *E.coli* O157:H7 with LOD 14 CFU/mL. The proposed fluorescent biosensor used the double-layer channel with the immune magnetic nanoparticles (MNPs) for specific separation and efficient concentration of the target bacteria, and the immune CdSe/ZnS QDs with a portable optical system for quantitative detection of the bacteria. Initially, the bacteria were captured by the immune MNPs in the channel at the presence of the high gradient magnetic fields (HGMFs) to form the MNP-bacteria complexes. Then, the immune QDs were used to react with the target bacteria to form the MNP-bacteria-QDs complexes in the channel. Finally, the enriched complexes were collected and detected using the portable optical system to obtain increase the fluorescence intensity for final determination of the *E.coli* O157:H7 cells in the sample. Wu et al. [118] has prepared modified ZnSe/ZnS QDs by 3-mercaptopropionic acid and established a rapid fluorescence method to detect the *E. coli* cells count by using MPA-ZnSe/ZnS QDs as a fluorescence probe. The fluorescence peak intensity increases with increasing cells count of bacteria. Compared with the traditional fluorescent detection methods, this one is more convenient and useful in the bacterial count determination with LOD 10^1^ CFU·mL^−1^.

In addition to bacteria, QDs are also used for virus detection. Jimenez et al. reported work which was focused on the development of a nano-system for simultaneous identification of HIV and HPV viruses with 1 nM of LOD. Their construction and characterization were carried doubt using magnetic glass particles (MGPs) which joined with target DNA oligonucleotides and the second part of the construction formed by the conjugation of red and green CdTe QDs with oligodeoxyribonucleotides complementary probe, derived from these two viruses, that encode respectively their capsid and oncoproteins. As a result, after the conjugating, the fluorescent intensity was slightly reduced in both cases [119].

## 8. Conclusions and Perspectives

Quantum dots have remarkable optical properties, which make them among the most useful nanomaterials [6]. They may be utilized in a wide range of applications, e.g., in new types of fluorescent probes and as active components of nanostructure-biomolecule complexes [122]. Various schemes for the application of optical transduction QDs have been successfully tested, allowing a wide range of detection, high selectivity and sensitivity in the tested samples. The development of analytical methods for the detection of various chemical or biological compounds allows the use of QDs in sensors for determining the presence of ions, molecules and pH changes. The results of discussed studies lead to the improvement of existing detection devices and the design of new detection devices that allow more sensitive and faster analysis. Quantum dots-based detection technologies can be adapted to precision medical technologies by overturning point-of-care (POC) and personalized diagnostics. This engineering can supply high-throughput and mobile diagnostic platforms for screening pathogens and toxins immediately in field and POC clinical settings. Several of these technologies tender multiplexing capacities for simultaneous examination of multiple analytes with unexpectedly high sensitivity that can notably lower costs and detection time. Nevertheless, a universal sensor for different types of medicinal/or, i.e., food samples, is a challenge because of the inherent complexity of biological samples. Evaluation with numerous of biological (i.e., food, body fluids) samples and comparison with well-established techniques may assist to direct this challenge.

Among further fields that could exploit some of the advantages of QDs are fluorescent immunosensors designed as integrated devices. Due to the relevant improvement on the execution of fluorescent immunosensors and recent advances in miniaturization processes, it is believed that in the near future small and advanced fluorescent mobile analytical platforms, which combine steps of the immunoassay pathway, will be available. In conclusion, there is potential for further investigations of QDs in multiplex detection, particularly via continued miniaturization and integration into lab-on-chip platforms.

## Figures and Tables

**Table 1:** QDs-based sensors for heavy metal ions determination.

Marker	Sensing Platform	Transduction Type	LOD *	References
Cu^2+^	CdS QDs	Change in fluorescence intensity	0.8 μM	[13]
Fe^3+^	CdTe QDs	Change in fluorescence intensity	14 nM	[57]
Hg^2+^	CdSe/ZnS QDs	Change in fluorescence intensity	15 nM	[58]
Pb^2+^	CdSe/CdS QDs	Change in fluorescence intensity	0.006 nM	[59]
Hg^2+^	TGA-CdTe QDs	FRET	20 nM	[60]
Pb^2+^	AuNPs & CA_CdTE QDs	FRET	30 ppb	[61]

* LOD—limit of detection

**Table 2:** QDs-based sensors for pH determination.

Marker	Sensing Platform	Transduction Type	References
pH	CdSe/ZnS-methacrylic acid QDs	FRET	[12]
pH in range 3–11	CdSe/ZnS- [1,3] oxazine QDs	Change in fluorescence intensity	[66]
pH	CdSe/ZnS-phosphine oxide NC	FRET	[67]
pH in range 4–6	CdTe-thioglycolic acid NC	Change in luminescence intensity	[68]
pH	CdTe QDs	Change in fluorescence intensity	[69]
Urea	CdSe/ZnS-mercaptosuccinig acid QDs	Change in fluorescence intensity	[70]

**Table 3:** QDs-based sensors for proteins determination.

Marker	Sensing Platform	Transduction Type	LOD	References
Protein kinases	CdTe QDs	Change in fluorescence intensity	5.0 fM	[19]
C-reactive protein	CdSe/ZnS QDs	Change in fluorescence intensity	0.47 mU·μL^−1^	[71]
Exonuclease III	DNA-ZnS:Mn^2+^	Change in fluorescence intensity	2.8 ng/mL	[72]
Micrococcal nuclease	CdSe/CdS	FRET	0.06 µ·mL^−1^	[73]
Caspase	CdSe/ZnS	FRET	20 pM	[74]
Thrombin	PbS QDs	FRET	1 nM	[75]

**Table 4:** QDs-based sensors for sugars determination.

Marker	Sensing Platform	Transduction Type	LOD	References
Glucose	CdSe/ZnS-TGA	Change in fluorescence intensity	-	[77]
Glucose	QDs-ConA-β-CDs-AuNPs	FRET	50 nM	[78]
Glucose and fructose	CdSe/ZnS QDs	Change in fluorescence intensity	1 μM	[79]
Glucose	Mn-ZnS QDs	Change in phosphorescence intensity	3 μM	[80]
Glucose	CdTe QDs	Change in fluorescence intensity	0.10 μM	[81]

**Table 5:** QDs-based sensors for nucleic acids determination.

Marker	Sensing Platform	Transduction Type	LOD	References
DNA in urine	CdTe QDs	Change in fluorescence intensity	3 ng·mL^−1^	[83]
Mitoxantrone and ribonucleic acid	CdTe QDs	Change in fluorescence intensity	0.1 ng/µL	[84]
dsDNA	CdTe QDs	FRET	-	[85]
DNA, micro-RNA	CdTe/CDs QDs	Change in fluorescence intensity	1 fM	[86]

**Table 6:** QDs-based sensors for neurotransmitters determination.

Marker	Sensing Platform	Transduction Type	LOD	References
Dopamine	QD@SiO_2_	FRET	12.5 nM	[89]
Dopamine	SnSe QDs	Photoelectrochemical assay	3 nM	[90]
Norepinephrine	CdTe@SiO_2_@MIP	Change in fluorescence intensity	8 nM	[91]
Serotonin	ZnS QDs	Change in fluorescence intensity	0.69 ng·mL^−1^	[30]
Acetylcholine	CdSe/ZnS	Change in fluorescence intensity	-	[92]

**Table 7:** QDs-based sensors for pesticides determination.

Marker	Sensing Platform	Transduction Type	LOD	References
Glyphosate	CdTe QDs	Change in fluorescence intensity	0.5 μg·mL^−1^	[97]
Trichlorfon	CdSe/ZnS	Change in fluorescence intensity	9.0 μg·L^−^^1^	[98]
Organophosphorus	CdTe-TGA QDs	Change in fluorescence intensity	0.68 nM	[99]
Thiram	ZnS:Mn^2+^	Change in phosphorescence intensity	25 nM	[100]
Doxycycline	TGA/CdTe QDs	Change in fluorescence intensity	1.1 × 10^−7^ mol·L^−1^	[101]
Paraquat	CdSe/ZnS QDs	Change in fluorescence intensity	3.0 ng·L^−1^	[102]

**Table 8:** QDs-based biosensors for toxins determination.

Marker	Sensing Platform	Transduction Type	LOD	References
Saxitoxin	CdS/CdSe/ZnS QDs	Change in fluorescence intensity	0.3 μg·kg^−1^	[103]
Botulinum neurotoxin	Carboxyl QDs	FRET	0.2 ng·mL^−1^	[104]
Cholera toxin, ricin, shiga-like toxin 1, staphylococcal enterotoxin B	CdSe/ZnS QDs	Change in photoluminescence intensity	-	[105]
Ochratoxin A	CdS QDs	Square-Wave Stripping Voltammetry	0.2 pg·mL^−^^1^	[106]

**Table 9:** QDs-based sensors for volatile substances determination.

Marker	Sensing Platform	Transduction Type	LOD	References
Ammonia gas	PbS QDs/TiO_2_ NTARs	Change in fluorescence intensity	2 ppm	[109]
Methanol	CdS QDs	Photoelectrochemical assay	0.14 µg·L^−1^	[110]
Acetone	Mn^2+^-ZnS QDs	Change in room temperature phosphorescence intensity	0.2 mg·L^−1^	[111]
Ethanol, 2-propanol, acetone	CdTe QDs	Change in photoluminescence intensity	-	[108]

**Table 10:** QDs-based sensors for vitamins determination.

Marker	Sensing Platform	Transduction Type	LOD	References
Vitamin E	CdSe/ZnS QDs, GOx	Change in fluorescence intensity	3.5 nM	[112]
Folic acid	ZnS:Cu/ZnS-MPA QDs	Change in fluorescence intensity	11 μM	[113]
Vitamin B_12_	CDs	FRET	0.1 μg·mL^−1^	[114]
Vitamin B_6_	CdTe-TGA QDs	Change in fluorescence intensity	-	[115]
Ascorbic Acid	CdS-diphenylcarbazide QDs	FRET	2 nM	[116]

**Table 11:** QDs-based sensors for bacteria and viruses determination.

Marker	Sensing Platform	Transduction Type	LOD	References
*E. coli*	CdSe/ZnS QDs	Change in fluorescence intensity	1.4 × 10^1^ CFU·mL^−^^1^	[117]
*E. coli*	MPA-ZnSe/ZnS QDs	Change in fluorescence intensity	10^1^ CFU·mL^−1^	[118]
HIV and HPV	CdTe QDs	Change in fluorescence intensity	100 nM	[119]
*E. coli*	CdSe/ZnS QDs	Change in fluorescence intensity	2.08 × 10^7^ CFU·mL^−1^	[120]
*E. coli*	CdSe/ZnS QDs	Change in fluorescence intensity	2.3 CFU·mL^−1^	[121]

## References

[1] Luo Z., Chen Y., Wu S.-T. (2013). Wide color gamut LCD with a quantum dot backlight. Opt. Express.

[2] Song H.-J., Jeong B.G., Lim J., Lee D.C., Bae W.K., Klimov V.I. (2018). Performance Limits of Luminescent Solar Concentrators Tested with Seed/Quantum-Well Quantum Dots in a Selective-Reflector-Based Optical Cavity. Nano Lett..

[3] Liu H., Lia S., Chen W., Wang D., Lia C., Wu D., Hao J., Zhoua Z., Wang X., Wang K. (2018). Scattering enhanced quantum dots based luminescent solar concentrators by silica microparticles. Sol. Energy Mater. Sol. Cells.

[4] Zhang Y., Clapp A. (2011). Overview of Stabilizing Ligands for Biocompatible Quantum Dot Nanocrystals. Sensors.

[5] Larson D.R., Zipfel W.R., Williams R.M., Clark S.W., Bruchez M.P., Wise F.W. (2003). Water-Soluble Quantum Dots for Multiphoton Fluorescence Imaging in Vivo. Science.

[6] Frasco M.F., Chaniotakis N. (2009). Semiconductor Quantum Dots in Chemical Sensors and Biosensors. Sensors.

[7] Medintz I.L., Uyeda H.T., Goldman E.R., Mattoussi H. (2005). Quantum dot bioconjugates for imaging, labelling and sensing. Nat. Mater..

[8] Sapsford K.E., Algar R.W., Berti L., Gemmill K.B., Casey B.J., Oh E., Stewart M.H., Medintz I.L. (2013). Functionalizing Nanoparticles with Biological Molecules: Develpoing Chemistries that Facilitate Nanotechnology. Chem. Rev..

[9] Foubert A., Beloglazova Rajkovic A., Sas B., Madder A., Goryacheva I.Y., Saeger S.D. (2016). Bioconjugation of quantum dots: Review & impact on future application. Trends Anal. Chem..

[10] Hardman R. (2006). A Toxycologic Review of quantum Dots: Toxicity Depends on Physicochemical and Environmental Factors. Environ. Health Perspect..

[11] Lovrić J., Bazzi H.S., Cuie Y., Fortin G.R., Winnik F.M., Maysinger D. (2005). Differences in subcellular distribution and toxicity of green and red emitting CdTe quantum dots. J. Mol. Med..

[12] Liu Y.S., Sun Y., Vernier P.T., Liang C.H., Chong S.Y.C., Gundersen M.A. (2007). pH-sensitive photoluminescence of CdSe/ZnSe/ZnS quantum dots in human ovarian cancer cells. J. Phys. Chem. C.

[13] Chen Y., Rosenzweig Z. (2002). Luminescent CdS quantum dots as selective ion probes. Anal. Chem..

[14] Cissell K.A., Campbell S., Deo S.K. (2008). Rapid, single-step nucleic acid detection. Anal. Bioanal. Chem..

[15] Dabbousi B.O., Rodriguez-Viejo J., Mikulec F.V., Heine J.R., Mattoussi H., Ober R., Jensen K.F., Bawendi M.G. (1997). (CdSe)ZnS Core-Shell Quantum DotsL Synthesis and Characterization of a Size Series of Highly Luminescent Nanocrystallites. J. Phys. Chem. B.

[16] Wang X., Qu L., Zhang J., Peng X., Xiao M. (2003). Surface-related emission in highly luminescent CdSe QDs. Nano Lett..

[17] Resch-Genger U., Grabolle M., Cavaliere-Jaricot S., Nitschke R., Nann T. (2008). Quantum dots versus organic dyes as fluorescent labels. Nat. Methods.

[18] Goldman E.R., Medintz I.L., Mattoussi H. (2006). Luminescent quantum dots in immunoassays. Anal. Bioanal. Chem..

[19] Xu X., Liu X., Nie Z., Pan Y., Guo M., Yao S. (2011). Label-Free Fluorescent Detection of Protein Kinase Activity Based on the Aggregation Behavior of Unmodified Quantum Dots. Anal. Chem..

[20] Algar R.W., Tavares A.J., Krull U.J. (2010). Beyond labels: A review of the application of quantum dots as integrated components of assays, bioprobes, and biosensors utilizing optical transduction. Anal. Chim. Acta.

[21] Gründler P. (2007). Chemical Sensors: An Introduction for Scientists and Engineers.

[22] Nowak Wenger W., Bates F.S., Aydil E.S. (2017). Functionalization of Cadmium Selenide Quantum Dots with Poly(ethylene glycol): Ligand Exchange, Surface Coverage, and Dispersion Stability. Langmuir.

[23] Zhu H., Hu M.Z., Shao L., Yu K., Dabestani R., Zaman Md B., Liao S. (2014). Synthesis and Optical Properties of Thiol Functionalized CdSe/ZnS (Core/Shell) Quantum Dots by Ligand Exchange. J. Nanomater..

[24] Liu X., Luo Y. (2014). Surface Modifications Technology of Quantum Dots Based Biosensors and Their Medical Applications. Chin. J. Anal. Chem..

[25] Blanco-Canosa J.B., Wu M., Susumu K., Petryayeva E., Jennings T.L., Dawson P.E., Algar W.R., Medintz I.L. (2014). Recent progress in the bioconjugation of quantum dots. Coord. Chem. Rev..

[26] Zhou J., Liu Y., Tang J., Tang W. (2017). Surface ligands engineering of semiconductor quantum dots for chemosensory and biological applications. Mater. Today.

[27] Nsibande S.A., Forbes P.B.C. (2016). Fluorescence detection of pesticides using quantum dot materials—A review. Anal. Chim. Acta.

[28] Walia S., Acharya A. (2014). Fluorescent cadmium sulfide nanoparticles for selective and sensitive detection of toxic pesticides in aqueous medium. J. Nanopart. Res..

[29] De Bastida G., Arregui F.J., Javier Goicoechea J., Matias I.R. (2006). Quantum Dots-Based Optical Fiber Temperature Sensors Fabricated by Layer-by-Layer. IEEE Sens. J..

[30] Wang Z., Zhang Y., Zhang B., Lu X. (2018). Mn^2+^ doped ZnS QDs modified fluorescence sensor based on molecularly imprinted polymer/sol-gel chemistry for detection of Serotonin. Talanta.

[31] Jiang H., Ju H. (2007). Electrochemiluminescence Sensors for Scavengers of Hydroxyl Radical Based on Its Annihilation in CdSe Quantum Dots Film/Peroxide System. Anal. Chem..

[32] Jie G., Zhang J., Wang D., Cheng C., Chen H.-Y., Zhu J.-J. (2008). Electrochemiluminescence Immunosensor Basedon CdSe Nanocomposites. Anal. Chem..

[33] Shang L., Zhang L., Dong S. (2009). Turn-on fluorescent cyanide sensor based on copper ion-modified CdTe quantum dots. Analyst.

[34] Wang L.Y., Wang L., Gao F., Yu Z.Y., Wu Z.M. (2002). Application of functionalized CdS nanoparticles as fluorescence probe in the determination of nucleic acid. Analyst.

[35] Kavosia B., Navaee A., Salimi A. (2018). Amplified fluorescence resonance energy transfer sensing of prostate specific antigen based on aggregation of CdTe QDs/antibody and aptamer decoratedof AuNPs-PAMAM dendrimer. Luminescence.

[36] Anikeeva P.O., Madigan C.F., Halpert J.E., Bawendi M.G., Bulović V. (2008). Electronic and excitonic processes in light-emitting devices based on organic materials and colloidal quantum dots. Phys. Rev. B.

[37] Van Sark WGJ H.M., Frederix PL T.M., Bol A.A., Gerritsen H.C., Meijerink A. (2002). Blueing, Bleaching, and Blinking of Single CdSe/ZnS Quantum Dots. Chemphyschem.

[38] Wang L.Y., Kan X.W., Zhang M.C., Zhu C.Q., Wang L. (2002). Fluorescence for the determination of protein with functionalized nano-ZnS. Analyst.

[39] Loura L.M.S., Prieto M. (2011). FRET in membrane biophysics: An overview. Front. Physiol..

[40] Clapp A.R., Medintz I.L., Mattoussi H. (2006). Förster Resonance Energy Transfer Investigations Using Quantum-Dot Fluorophores. ChemPhysChem.

[41] Miyawaki A. (2003). Visualization of the spatial and temporal dynamics of intracellular signaling. Dev. Cell.

[42] Miyawaki A. (2008). Green Fluorescent Protein Glows Gold. Cell.

[43] Lasota S., Baster Z., Witko T., Zimoląg E., Sroka J., Rajfur Z., Madeja Z. (2017). Zastosowanie biosensorów typu FRET w badaniach mikroskopowych procesu migracji komórkowej. Postępy Biochemii.

[44] Clapp A.R., Medintz I.L., Mauro J.M., Fisher B.R., Bawendi M.G., Mattoussi H. (2004). Fluorescence resonance energy transfer between quantum dot donors and dye-labeled protein acceptors. J. Am. Chem. Soc..

[45] Patolsky F., Gill R., Weizmann Y., Mokari T., Banin U., Willner I. (2003). Lighting-Up the Dynamics of Telomerization and DNA Replication by CdSe-ZnS Quantum Dots. J. Am. Chem. Soc..

[46] Coto-García A.M., Sotelo-González E., Fernández-Argüelles M.T., Pereiro R., Costa-Fernández J.M., Sanz-Medel A. (2011). Nanoparticles as fluorescent labels for optical imaging and sensing in genomics and proteomics. Anal. Bioanal. Chem..

[47] Freeman R., Girsh J., Willner I. (2013). Nucleic Acid/Quantum Dots (QDs) Hybrid Systems for Optical and Photoelectrochemical Sensing. ACS Appl. Mater. Interfaces.

[48] Zhang H., Zhang L., Liang R.-P., Huang J., Qiu J.-D. (2013). Simultaneous Determination of Concanavalin A and Peanut Agglutinin by Dual-Color Quantum Dots. Anal. Chem..

[49] Waggonera P.S., Craighead H.G. (2007). Micro- and nanomechanical sensors for environmental, chemical, and biological detection. Lab Chip.

[50] Mo G., He X., Zhou C., Ya D., Feng J., Yu C., Deng B. (2018). Sensitive detection of hydroquinone based on electrochemiluminescence energy transfer between the exited ZnSe quantum dots and benzoquinone. Sens. Actuator B Chem..

[51] Yao J., Li L., Li P., Yang M. (2017). Quantum dots: From fluorescence to chemiluminescence, bioluminescence, electrochemiluminescence, and electrochemistry. Nanoscale.

[52] Haram S.K., Quinn B.M., Bard A.J. (2001). Electrochemistry of CdS Nanoparticles: A Correlation between Optical and Electrochemical Band Gaps. J. Am. Chem. Soc..

[53] Huang H., Zhu J.J. (2013). The electrochemical applications of quantum dots. Analyst.

[54] Liu Y., Zhu L., Kong J., Yang P., Liu B. (2013). A quantum dots-based electrochemical assay towards the sensitive detection of tumor cells. Electrochem. Commun..

[55] Xu Y., Liu J., Gao C., Wang E. (2014). Applications of carbon quantum dots in electrochemiluminescence: A mini review. Electrochem. Commun..

[56] Bertoncello P., Ugo P. (2017). Recent Advances in Electrochemiluminescence with Quantum Dots and Arrays of Nanoelectrodes. ChemElectroChem.

[57] Zhou M., Guo J., Yang C. (2018). Ratiometric fluorescence sensor for Fe3+ ions detection based on quantum dot-doped hydrogel optical fiber. Sens. Actuator B Chem..

[58] Li H., Zhang Y., Wang X., Xiong D., Bai Y. (2007). Calixarene capped quantum dots as luminescent probes for Hg^2+^ ions. Mater. Lett..

[59] Zhao Q., Rong X., Ma H., Tao G. (2013). Dithizone functionalized CdSe/CdS quantum dots as turn-on fluorescent probe for ultrasensitive detection of lead ion. J. Hazard. Mater..

[60] Li J., Mei F., Li W.Y., He X.W., Zhang Y.K. (2008). Study on the fluorescence resonance energy transfer between CdTe QDs and butylrhodamine B in the presence of CTMAB and its application on the detection of Hg(II). Spectrochim. Acta Part A.

[61] Wang X., Guo X. (2009). Ultrasensitive Pb^2+^ detection based on fluorescence resonance energy transfer (FRET) between quantum dots and gold nanoparticles. Analyst.

[62] Wang C., Huang Y., Jiang K., Humphrey M.G., Zhang C. (2016). Dual-emitting quantum dot/carbon nanodot-based nanoprobe for selective and sensitive detection of Fe3+ in cells. Analyst.

[63] Brahim N.B., Mohamed N.B.H., Echabaane M., Haouari M., Chaâbane R.B., Negrerie M., Ouadaa H.B. (2015). Thioglycerol-functionalized CdSe quantum dots detecting cadmium ions. Sens. Actuators B Chem..

[64] Yao J., Zhang K., Zhu H., Ma F., Sun M., Yu H., Sun J., Wang S. (2013). Efficient ratiometric fluorescence probe based on dual-emission quantum dots hybrid for on-site determination of copper ions. Anal. Chem..

[65] Xu H., Zhang K., Liu Y., Xie M. (2017). Visual and fluorescent detection of mercury ions by using a dually emissive ratiometric nanohybrid containing carbon dots and CdTe quantum dots. Microchim. Acta.

[66] Tomasulo M., Yildiz I., Kaanumalle S.L., Raymo F.M. (2006). pH-Sensitive Ligand for Luminescent Quantum Dots. Langmuir.

[67] Snee P.T., Somers R.C., Nair G., Zimmer J.P., Bawendi M.G., Nocera D.G. (2006). A Ratiometric CdSe/ZnS Nanocrystals pH Sensor. J. Am. Chem. Soc..

[68] Susha A.S., Javier A.M., Parak W.J., Rogach A.L. (2006). Luminescent CdTe nanocrystals as ion probes and pH sensors in aqueous solutions. Colloids Surf. A.

[69] Wang Y., Ye C., Zhu Z.H., Hu Y.Z. (2008). Cadmium telluride quantum dots as pH-sensitive probes for tiopronin determination. Anal. Chim. Acta.

[70] Huang C.P., Li Y.L., Chen T.M. (2007). A highly sensitive system for urea detection by using CdSe/ZnS core-shell quantum dots. Biosens. Bioelectron..

[71] Lv Y., Wu R., Feng K., Li J., Mao Q., Yuan H., Shen H., Chai X., Li L.S. (2017). Highly sensitive and accurate detection of C-reactive protein by CdSe/ZnS quantum dot-based fluorescence-linked immunosorbent assay. J. Nanobiotechnol..

[72] Zhang C., Ding C., Zhou G., Xue Q., Xian Y. (2017). One-step synthesis of DNA functionalized cadmium-free quantum dots and its application in FRET-based protein sensing. Anal. Chim. Acta.

[73] Huang S., Xiao Q., He Z.K., Liu Y., Tinnefeld P., Su X.R., Peng X.N. (2008). A high sensitive and specific QDs FRET bioprobe for MNase. Chem. Commun..

[74] Boeneman K., Mei B.C., Dennis A.M., Bao G., Deschamps J.R., Mattoussi H., Medintz I.L. (2009). Sensing caspase 3 activity with quantum dot-fluorescent protein assemblies. J. Am. Chem. Soc..

[75] Choi J.H., Chen K.H., Strano M.S. (2006). Aptamer-capped nanocrystal quantum dots: A new method for label-free protein detection. J. Am. Chem. Soc..

[76] Hu J., Wang Z.Y., Li C., Zhang C. (2017). Advances in single quantum dot-based nanosensors. Chem. Commun..

[77] Saran A.D., Sadawana M.M., Srivastava R., Bellare J.R. (2011). An optimized quantum dot-ligand system for biosensing applications: Evaluation as a glucose biosensor. Colloids Surf. A.

[78] Tang B., Cao L., Xu K., Zhuo L., Ge J., Li Q., Yu L. (2008). A New Nanobiosensor for Glucose with High Sensitivity and Selectivity in Serum Based on Fluorescence Resonance Energy Transfer (FRET) between CdTe Quantum Dots and Au Nanoparticles. Chem. Eur. J..

[79] Riedel M., Sabir N., Scheller F.W., Parak W.J., Lisdat F. (2017). Connecting quantum dots with enzymes: Mediator-based approaches for the light-directed read-out of glucose and fructose oxidation. Nanoscale.

[80] Wu P., He Y., Wang H.F., Yan X.P. (2010). Conjugation of Glucose Oxidase onto Mn-Doped ZnS Quantum Dots for Phosphorescent Sensing of Glucose in Biological Fluids. Anal. Chem..

[81] Cao L., Ye J., Tong L., Tang B. (2008). A new route to the considerable enhancement of glucose oxidase (GOx) activity: The simple assembly of a complex from CdTe quantum dots and GOx and its glucose sensing. Chem. Eur. J..

[82] Hua M., Tiana J., Lua H.T., Weng L.X., Wang L.H. (2010). H_2_O_2_-sensitive quantum dotsfor the label-free detection of glucose. Talanta.

[83] Nejdl L., Zelnickova J., Vaneckova T., Hynek D., Adam V., Vaculovicova M. (2018). Rapid preparation of self-assembled CdTe quantum dots used for sensing of DNA in urine. New J. Chem..

[84] Mohammadinejad A., Es’haghi Z., Abnous K., Mohajeri S.A. (2017). Tandem determination of mitoxantrone and ribonucleic acid using mercaptosuccinic acid-capped CdTe quantum dots. J. Lumin..

[85] Dai Z., Zhang J., Dong Q., Guo N., Xu S., Sun B., Bu Y. (2007). Adaptation of Au Nanoparticles and CdTe Quantum Dots in DNA Detection. Chin. J. Chem. Eng..

[86] Su S., Fan J., Xue B., Yuwen L., Liu X., Pan D., Fan C., Wang L. (2014). DNA-Conjugated Quantum Dot Nanoprobe for High-Sensitivity Fluorescent Detetction of DNA and micro-RNA. ACS Appl. Mater. Interfaces.

[87] Pei X., Yin H., Lai T., Zhang J., Liu F., Xu X., Li N. (2018). Multiplexed Detection of Attomoles of Nucleic Acids Using Fluorescent Nanoparticle Counting Platform. Anal. Chem..

[88] Ghasemi F., Hormozi-Nezhad M.R., Mahmoudi M. (2016). Identification of catecholamine neurotransmitters using fluorescence sensor array. Anal. Chim. Acta.

[89] Wang B., Chen M., Zhang H., Wen W., Zhang X., Wang S. (2017). A simple and sensitive fluorometric dopamine assay based on silica-coated CdTe quantum dots. Microchim. Acta.

[90] Hun X., Wang S., Mei S., Qin H., Zhang H., Luo X. (2017). Photoelectrochemical dopamine sensor based on a gold electrode modified with SnSe nanosheets. Microchim. Acta.

[91] Wei F., Wu Y., Xu G., Gao Y., Yang J., Liu L., Zhou P., Hu Q. (2014). Molecularly imprinted polymer based on CdTe@SiO2 quantum dots as a fluorescent sensor for the recognition of norepinephrine. Analyst.

[92] Jin T., Fujii F., Sakata H., Tamura M., Kinjo M. (2005). Amphiphilic p-sulfonatocalix[4]arene-coated CdSe/ZnS quantum dots for the optical detection of the neurotransmitter acetylcholine. Chem. Commun..

[93] Malecha K., Macioszczyk J., Słobodzian P. (2018). Application of microwave heating in ceramic-based microfluidic module. Microelectron. Int..

[94] Malecha K. (2016). The Implementation of Fluorescence-Based Detection in LTCC (Low-Temperature-Co-Fired-Ceramics) Microfluidic Modules. Int. J. Appl. Ceram. Technol..

[95] Golonka L.J. (2006). Technology and applications of Low Temperature Cofired Ceramic (LTCC) based sensors and microsystems. Bull. Pol. Acad. Sci. Tech. Sci..

[96] Baluta S., Malecha K., Zając D., Sołoducho J., Cabaj J. (2017). Dopamine sensing with fluorescence strategy based on low temperature co-fired ceramic technology modified with conducting polymers. Sens. Actuator B Chem..

[97] López J., Llorent-Martinez J., Ortega-Barrales P., Ruiz-Medina A. (2018). Multicommutated Flow System for the Determination of Glyphosate Based on Its Quenching Effect on CdTe-Quantum Dots Fluorescence. Food Anal. Methods.

[98] Liu Q., Jiang M., Ju Z., Qiao X., Xu Z. (2018). Development of direct competitive biomimetic immunosorbent assay basedon quantum dot label for determination of trichlorfon residues in vegetables. Food Chem..

[99] Kanagasubbulakshmi S., Kathiresan R., Kadirvelu K. (2018). Structure and physiochemical properties based interaction patterns of organophosphorous pesticides with quantum dots: Experimental and theoretical studies. Colloids Surf. A.

[100] Zhang C., Zhang K., Zhao T., Liu B., Wang Z., Zhang Z. (2017). Selective phosphorescence sensing of pesticide based on the inhibition of silver(I) quenched ZnS:Mn^2+^ quantum dots. Sens. Actuator B Chem..

[101] Tashkhourian J., Absalan G., Jafari M., Zare S. (2016). A rapid and sensitive assay for determination of doxycycline using thioglycolic acid-capped cadmium telluride quantum dots. Spectrochim. Acta Part A.

[102] Durán G.M., Contento A.M., Ríos Á. (2013). Use of Cdse/ZnS quantum dots for sensitive detection and quantification of paraquat in water samples. Anal. Chim. Acta.

[103] Sun A., Chai J., Xiao T., Shi X., Li X., Zhao Q., Li D., Chen J. (2018). Development of a selective fluorescence nanosensor based on molecularly imprinted-quantum dot optosensing materials for saxitoxin detection in shellfish samples. Sens. Actuator B Chem..

[104] Wang Y., Fry H.C., Skinner G.E., Schill K.M., Duncan T.V. (2017). Detection and Quantification of Biologically Active Botulinum Neurotoxin Serotypes A and B Using a Förster Resonance Energy Transfer-Based Quantum Dot Nanobiosensor. ACS Appl. Mater. Interfaces.

[105] Goldman E.R., Clapp A.R., Anderson G.P., Uyeda H.T., Mauro J.M., Medintz I.L., Mattoussi H. (2004). Multiplexed Toxin Analysis Using Four Colors of Quantum Dot Fluororeagents. Anal. Chem..

[106] Tong P., Zhao W.-W., Zhang L., Xua J.-J., Chen H.-Y. (2012). Double-probe signal enhancing strategy for toxin aptasensing based on rolling circle amplification. Biosens. Bioelectron..

[107] Ligler F.S., Taitt C.R., Shriver-Lake L.C., Sapsford K.E., Shubin Y., Golden J.P. (2003). Array biosensor for detection of toxins. Anal. Bioanal. Chem..

[108] Bakar N.A., Rahmi A., Umar A.A., Salleh M.M., Muhammad Yahaya M. (2011). Fluorescence Gas Sensor Using CdTe Quantum Dots Film to Detect Volatile Organic Compounds. Mater. Sci. Forum.

[109] Liua Y., Wanga L., Wanga H., Xionga M., Yanga T., Zakharova G.S. (2016). Highly sensitive and selective ammonia gas sensors based on PbS quantum dots/TiO2 nanotube arrays at room temperature. Sens. Actuator B Chem..

[110] Barroso J., Díez-Buitrago B., Saa L., Möller M., Briz N., Pavlov V. (2018). Specific bioanalytical optical and photoelectrochemical assays for detectionof methanol in alcoholic beverages. Biosens. Bioelectron..

[111] Sotelo-Gonzalez E., Fernandez-Argüelles M.T., Costa-Fernandez J.M., Sanz-Medel A. (2012). Mn-doped ZnS quantum dots for the determination of acetone by phosphorescence attenuation. Anal. Chim. Acta.

[112] Liu H., Fang G., Zhu H., Li C., Liu C., Wang S. (2013). A novel ionic liquid stabilized molecularly imprinted optosensing material based on quantum dots and graphene oxide for specific recognition of vitamin E. Biosens. Bioelectron..

[113] Geszke-Moritz M., Clavier G., Lulek J., Schneider R. (2012). Copper- or manganese-doped ZnS quantum dots as fluorescent probes for detecting folic acid in aqueous media. J. Lumin..

[114] Wang J., Wei J., Su S., Qiu J. (2015). Novel fluorescence resonance energy transfer optical sensors for vitamin B_12_ detection using thermally reduced carbon dots. New J. Chem..

[115] Sun J.F., Ren C.L., Liu L.H., Chen X.G. (2008). CdTe quantum dots as fluorescence sensor for the determination of vitamin B6 in aqueous solution. Chin. Chem. Lett..

[116] Ganiga M., Cyriac J. (2016). An ascorbic acid sensor based on cadmium sulphide quantum dots. Anal. Bioanal. Chem..

[117] Xue L., Zheng L., Zhan H., Jinc X., Lin J. (2018). An ultra sensitive fluorescent biosensor using high gradient magnetic separation and quantum dots for fast detection of foodborne pathogenic bacteria. Sens. Actuator B Chem..

[118] Wu P., Huang R., Li G., He Y., Chen C., Xiao W., Ding P. (2018). Optimization of Synthesis and Modification of ZnSe/ZnS Quantum Dots for Fluorescence Detection of *Escherichia coli*. J. Nanosci. Nanotechnol..

[119] Jimenez A.M.J., Moulick A., Richtera L., Krejcova L., Kalina L., Datta R., Svobodova M., Hynek D., Masarik M., Heger Z. (2018). Dual-color quantum dots-based simultaneous detection of HPV-HIV co-infection. Sens. Actuator B Chem..

[120] Hahn M.A., Tabb J.S., Krauss T.D. (2005). Detection of Single Bacterial Pathogens with Semiconductor Quantum Dots. Anal. Chem..

[121] Liu Y., Brandon R., Cate M., Peng X., Stony R., Johnson M. (2007). Detection of pathogens using luminescent CdSe/ZnS dendron nanocrystals and a porous membrane immunofilter. Anal. Chem..

[122] Hu P., Chen L., Kang X., Chen X. (2016). Surface Functionalization of Metal Nanoparticles by Conjugated Metal–Ligand Interfacial Bonds: Impacts on Intraparticle Charge Transfer. Acc. Chem. Res..

